# Manganese Nanomaterials: A Green Solution to Suppress *Xanthomonas oryzae* in Rice

**DOI:** 10.3390/plants14101540

**Published:** 2025-05-20

**Authors:** Yaqi Jiang, Yi Sun, Pingfan Zhou, Meng Tian, Yukui Rui

**Affiliations:** 1 Beijing Key Laboratory of Farmland Soil Pollution Prevention and Remediation, College of Resources and Environmental Sciences, China Agricultural University, Beijing 100193, China; jyq9797@163.com (Y.J.); 18732402125@163.com (Y.S.); zhoupingfan0516@163.com (P.Z.); 2 Department of Environmental Science and Engineering, University of Science and Technology of China, Hefei 230026, China; 3 Department of Plant Pathology, Ministry of Agriculture Key Laboratory of Pest Monitoring and Green Management, China Agricultural University, Beijing 100107, China; mengtian_2020@163.com; 4 Professor’s Workstation of Yuhuangmiao Town, China Agricultural University, Shanghe County, Jinan 250061, China; 5 Professor’s Workstation of Sunji Town, China Agricultural University, Shanghe County, Jinan 250061, China

**Keywords:** manganese oxide nanomaterials, manganese tetroxide nanomaterials, rice, *Xanthomonas oryzae*, nanopesticide, antibacterial, agriculture

## Abstract

Due to the environmental concerns surrounding widely used antimicrobial agents, the use of nanotechnology to suppress crop diseases has attracted increasing attention in the agricultural field. This paper investigated the inhibitory effects of manganese-based nanomaterials (NMs) on rice leaf blight. In vitro experiments showed that manganese oxide (MnO_2_) NMs and manganese tetroxide (Mn_3_O_4_) NMs directly inhibited *Xanthomonas oryzae* (Xoo) with a maximum OD value of 0.177, which was 11.5% lower than the control. In vivo experiments demonstrated that spraying MnO_2_ NMs and Mn_3_O_4_ NMs reduced the diseased leaf length to 22–28% and 25–26%, respectively. This is due to Mn-based NMs inducing enhanced plant resistance by increasing the activity of phenylalanine ammonia–lyase in rice leaves by 36–61%. Single particle inductively coupled plasma mass spectrometry showed that Mn_3_O_4_ NMs are more frequently retained as NMs in rice than MnO_2_ NMs, resulting in enhanced antimicrobial effects. Mn-based NMs exhibit strong antimicrobial activity and hold significant promise as alternatives for plant protection and agricultural applications; however, careful consideration must be given to their concentrations and application methods.

## 1. Introduction

In recent years, nanotechnology has developed rapidly and has been widely used in various industrial sectors and daily life [1,2]. As nanotechnology research continues, the utilization of nanomaterials (NMs) is having a significant impact on agriculture, and the sector continues to seek innovative strategies to improve productivity and sustainability. Recent studies have highlighted the potential of NMs to positively influence plant growth and resilience, opening new avenues for addressing agricultural challenges [3,4,5]. These advances are particularly important because climate change-induced environmental stresses (e.g., droughts, high temperatures and salinization) are becoming more frequent and severe, seriously threatening crop production and global food security [6]. Therefore, there is a need to find novel, efficient and environmentally sound technologies to enhance plant resistance and agricultural sustainability.

Currently, the management of plant diseases predominantly relies on chemical pesticides and fungicides. Despite their extensive use, the efficacy of these chemicals has progressively diminished due to the increasing resistance exhibited by pathogenic organisms [7]. This rise in resistance often prompts farmers to escalate chemical dosages, further exacerbating environmental degradation, leading to economic inefficiencies and posing risks to human health. Moreover, high doses of pesticides and fungicides have raised global concerns regarding their detrimental impact on ecosystems and biodiversity [8]. Recent trends towards sustainability have driven researchers to seek alternative approaches to reduce dependency on traditional chemical pesticides.

Among numerous plant pathogens, *Xanthomonas oryzae* pv. oryzae (Xoo) represents one of the most destructive bacterial pathogens affecting rice cultivation worldwide [9]. This pathogen induces bacterial leaf blight (BLB), a disease that severely impacts rice crops, leading to considerable yield reductions, economic losses, and threats to food security, especially in regions heavily reliant on rice as a staple crop [10,11]. Rice is a globally important staple food that provides nutrition and an economic source for a large portion of the world’s population, especially in developing regions [12]. BLB affects rice plants at every stage of growth, and yield losses due to this disease generally range from 20% to 30%, with severe outbreaks potentially causing losses of up to 80%, depending on the susceptibility of the plant, the stage of growth at the time of infection and environmental conditions [13,14]. Considering the severe consequences of BLB and the reduced effectiveness of chemical control due to the emergence of resistance, the development of innovative, sustainable and effective alternative strategies is imperative to safeguard rice yields and ensure global food security.

NMs have emerged as a promising solution, which provide efficient pathogen control with substantially reduced chemical input [15,16]. For example, certain NMs, such as silver (Ag) [17] and copper oxide (CuO) [18], demonstrate multifaceted antibacterial mechanisms, including physical disruption and chemical interactions, significantly reducing the risk of pathogens developing resistance compared to conventional chemical agent [19].

Recent studies have explored the use of NMs as effective agents against Xoo, the causative agent of bacterial leaf blight in rice, while also promoting plant growth. Biogenic Ag NMs synthesized from plant extracts demonstrated strong antimicrobial activity against Xoo, inhibiting bacterial growth [20]. Copper (Cu) NMs were also evaluated, revealing dual benefits that effectively suppressed Xoo and simultaneously promoted rice growth by enhancing root and shoot lengths, chlorophyll content, and overall plant health [21]. Additionally, zinc oxide (ZnO) NMs exhibited significant antibacterial activity against Xoo and other rice pathogens, while also acting as growth promoters [22]. Moreover, Elsharkawy et al. [23] explored how Ag and Cu-based nanoparticles stimulate plant immune responses, enhancing resistance to fungal infections and improving plant health and productivity. Schiavi et al. [5] emphasized the potential of NMs in sustainable agriculture, particularly in pest and disease management, where they activate plant immune systems, reducing reliance on traditional chemical pesticides. Tatulli et al. [24] investigated the effectiveness of copper and Zn-based NMs in controlling diseases, demonstrating their precision and efficiency in pest management while minimizing harm to non-target organisms. Similarly, Choudhary et al. [25] showed that NMs could significantly reduce bacterial disease incidence by inducing plant defense mechanisms, further supporting the potential of NMs as biological control agents.

Despite these promising results, most studies have focused on single types of nanomaterials and often evaluated them at specific concentrations, with limited investigation into the effects of varying concentrations or combinations of different nanomaterials. Therefore, this study aims to explore the effects of two manganese-based nanomaterials (Mn-based NMs) (MnO_2_ and Mn_3_O_4_) at different concentrations on antibacterial activity against Xoo in rice. The findings of this research could provide new insights into the practical applications of nanomaterials in agricultural disease management.

In response to this critical agricultural challenge, this study investigates the feasibility of using Mn-based NMs, specifically manganese oxide (MnO_2_) and manganese tetroxide (Mn_3_O_4_), as innovative agents against Xoo-induced diseases in rice. Mn-based NMs were chosen for several reasons. First, manganese (Mn) plays a critical role in plant physiological processes, including photosynthesis, nutrient assimilation, and enzyme activity [26]. It is an essential micronutrient that is required to maintain plant growth and vigor under stressful conditions [27]. Secondly, available evidence suggests that MnO_2_ and Mn_3_O_4_ NMs have strong antimicrobial activity against a wide range of plant pathogens and do not produce significant phytotoxicity, even when applied at relatively high concentrations [28,29]. Mn-based NMs are distinct from traditional manganese salts (e.g., MnSO_4_ and MnCl_2_) due to their unique nanostructural characteristics [30], such as higher surface area and reactivity, which enhance their antimicrobial effects. These NMs, owing to their nanoscale size, exhibit greater surface interaction with plant and pathogen cells, leading to more efficient pathogen control [31]. Thirdly, these Mn-based NMs have been reported to increase the activity of antioxidant enzymes in plants, thereby enhancing their resistance to oxidative stress caused by diseases and environmental stresses [32]. Therefore, Mn-based NMs have the potential dual benefit of simultaneously controlling bacterial pathogens and enhancing the inherent ability of plants to withstand stresses.

The antimicrobial efficacy of Mn-based NMs on Xoo will be evaluated through growth inhibition and pathogen survival tests. The study also assesses the biological and physiological responses of rice, including diseased area, biomass, chlorophyll content, antioxidant enzyme activities and nutrient uptake. These analyses enhance our understanding of plant–microbe interactions and the mechanisms behind observed phenotypic and biochemical changes. This research demonstrates Mn-based NMs’ potential to control rice diseases while promoting plant health, aligning with sustainable development goals. It offers insights for reducing reliance on chemical pesticides and supports the broader application of nanotechnology in agriculture, contributing to eco-friendly, innovative strategies for food security and environmental protection.

## 2. Materials and Methods

### 2.1. Characterization of Mn-Based NMs

MnO_2_ NMs and Mn_3_O_4_ NMs were purchased from Shanghai Pantian NMs Co., Ltd., Shanghai, China (20–30 nm, purity > 99.9%). All other chemical reagents utilized in the experiment were obtained from Sinopharm Group (Beijing, China). The morphology and particle size of the NMs were detected by transmission electron microscopy (TEM-2100, JEOL Ltd., Tokyo, Japan). The image acquisition settings were as follows: accelerating voltage: 200 kV; magnification: 50,000× (scale 500 nm) and 100,000× (scale 200 nm). Details are shown in Table S1.

### 2.2. Cultivation of Rice Seedlings

Hybrid rice seeds (*Y Liangyou* 900) were purchased from the Chinese Academy of Agricultural Sciences (Beijing, China). After sterilizing the seeds for 30 min in a 10% hydrogen peroxide solution, they were washed in distilled water. The filter paper was then put on a sterile petri dish and the correct amount of distilled water was poured over it to keep it moist. Seeds were germinated in darkness for 48 h and then cultured for 4 d under conditions of 25 °C and 75% humidity. Six uniformly grown rice seedlings were selected, fixed onto sponges, and placed into 250 mL vials filled halfway with Kimura B nutrient solution (pH 5.5; Table S2). The seedlings were then cultured for an additional 7 d under conditions of 25 °C, 75% humidity, and a photoperiod of 16 h light and 8 h dark, after which they were used for subsequent in vivo and in vitro experiments.

### 2.3. In Vitro Experiments on Xoo

We conducted the experiment according to the methods of Li et al. [33] with some modifications. The nutrient broth (NB) medium was configured according to Table S2. Rice leaf blight bacterium *Xanthomonas oryzae pv. Oryzae strain* PXO99A was inoculated onto NA solid medium and incubated at 28 °C for 3–5 d. A small amount of PXO99A was collected and added to 5 mL of NA liquid medium, along with 5 μL of cephalosporin antibiotic. The bacteria were incubated in a shaker at 28 °C and 200 rpm for 24 h. The bacterial suspension was diluted with deionized water to reach OD600 = 0.1. MnO_2_ NMs and Mn_3_O_4_ NMs were added to the diluted bacterial suspension to reach a final concentration of 5, 25, or 50 µg/mL. The suspension was incubated for an additional 24 h. OD600 was measured using a UV spectrophotometer to determine the growth of bacteria.

### 2.4. In Vitro Experiments on Xoo-Infected Rice Leaves

The NB medium was sterilized in an autoclave at 121 °C for 2 h. Fresh leaves were cut from 4-wk-old rice seedlings, rinsed 3 times with sterile water, and sterilized by immersion in 2% sodium hypochlorite for 10 min. After that, 3 wounds were evenly created on the leaf veins with a sterile needle and each wound was inoculated with 10 µL of the bacterial (OD600 = 0.1). Twenty-four hours after inoculation, MnO_2_ NMs and Mn_3_O_4_ NMs (5, 25, or 50 µg/mL) were dropped onto the wounds using a pipette. The concentrations were chosen for this experiment according to previous research [34]. Changes in leaf wounds were observed and recorded after 7 d of incubation in an artificial chamber at 26 °C.

### 2.5. In Vivo Investigation of Antibacterial Activity of MnO_2_ and Mn_3_O_4_ NMs

MnO_2_ NMs and Mn_3_O_4_ NMs at 5, 25, and 50 µg/mL were evaluated for in vivo inhibition of the Xoo strain PXO99A. At the fourth leaf stage, leaves were inoculated with Xoo strain PXO99A or double distilled water by leaf clipping [35,36]. The rice was treated by foliar spray according to the method of Yasmin et al. [37] with MnO_2_ NMs and Mn_3_O_4_ NMs, post-inoculation after 24 h. The control for the disease treatment group was inoculated and sprayed with deionized water, and the control for the healthy group was not inoculated but was sprayed with deionized water. Each treatment had 3 replicates. The length and fresh biomass of roots and shoots were measured immediately after harvest. The percentage of disease leaf length (%DLL) was assessed 28 d post-inoculation by measuring the lesion leaf length relative to the total length of the leaf. The inhibition of Xoo compared to the untreated control was measured as the reduction in the average bacterial wilt length on the treated leaves using the following equation:

(1) $$\%\;\mathrm{Diseased}\;\mathrm{leaf}\;\mathrm{length}\;(\%\mathrm{DLL})=\frac{\mathrm{Total}\;\mathrm{lesion}\;\mathrm{length}\;\mathrm{of}\;\mathrm{the}\;\mathrm{test}\;\mathrm{sample}}{\mathrm{Total}\;\mathrm{leaf}\;\mathrm{length}\;\mathrm{of}\;\mathrm{the}\;\mathrm{test}\;\mathrm{sample}}\times 100$$

### 2.6. Leaf Contact Angle Measurement

The static contact angles of the Mn-based NMs on target leaves were measured using a contact angle measuring instrument (JC2000D, Zhongchen Digital Technic Apparatus Co., Ltd., Shanghai, China) (*n* = 3).

### 2.7. Antioxidant System of Rice After Exposure to MnO_2_ and Mn_3_O_4_ NMs

Fresh roots and shoots after 28 d post-inoculation were briefly homogenized in cold phosphate-buffered saline (PBS, pH 7.4) supplemented with 0.02 M Tris-HCl. The homogenate was centrifuged at 4000 rpm for 10 min at 4 °C, and the supernatant was carefully collected for subsequent enzyme assays. Measurements for peroxidase (POD), polyphenol oxidase (PPO), phenylalanine ammonia-lyase (PAL), and total phenolic content were conducted using assay kits of A084-3-1, A136-1-1, A137-1-1 and A143-1-1, respectively, following the manufacturer’s instructions of Nanjing Jiangcheng company, Nanjing, China. Each treatment was analyzed with 6 biological replicates.

For the POD activity assay, 0.1 mL of the prepared supernatant was combined with 2.4 mL of reagent A, 0.3 mL of reagent B, and 0.2 mL of reagent C. The reaction mixture was incubated in a 37 °C water bath for 30 min, after which 1.0 mL of reagent D was added. Following centrifugation at 3500 rpm for 10 min, absorbance readings of the supernatant were recorded at 420 nm.

To assess PPO activity, 0.15 mL of supernatant was mixed with 0.6 mL of reagent II and 0.15 mL of reagent III. This reaction was incubated at 37 °C for 30 min, then heated to above 90 °C for 5 min to terminate the reaction. The mixture was cooled rapidly under running water, centrifuged at 10,000 rpm for 10 min, and the absorbance of the resulting supernatant was measured at 420 nm.

To assess PAL activity, a mixture containing 0.04 mL of supernatant, 1.48 mL of reagent II and 0.4 mL of reagent III was placed in a 37 °C water bath for 30 min and then 0.4 mL of reagent IV was added. The absorbance of the supernatant was measured at 290 nm after standing for 10 min.

For the total phenolic content assay, the supernatant containing 0.05 mL was mixed with 0.25 mL of reagent I for 2 min and then 0.25 mL of reagent II was added to the mixture, as well as 0.45 mL of distilled water. The absorbance of the supernatant was measured at 760 nm after 10 min of standing.

The supernatants of the tissues treated with these different reagents were measured at different absorbance values using an enzyme-linked immunosorbent assay (ELISA) reader.

### 2.8. The Transfer and Transformation of MnO_2_ and Mn_3_O_4_ NMs in Rice

Rice shoot and root samples were incubated with 5% Macerozyme R-10 at 37 °C with shaking for 24 h. After enzymatic digestion, the samples were diluted 100-fold using ultrapure water and subsequently analyzed using single particle inductively coupled plasma mass spectrometry (sP-ICP-MS) with an Agilent 7900 ICP-MS (Agilent, Santa Clara, CA, USA). Instrument parameters followed a modified protocol based on the method reported by Tan et al. [38]. Particle size distribution, particle concentration, and dissolved Mn ion concentration were measured simultaneously for each sample. Analyses were conducted using a dwell time of 100 µs and a total sampling duration of 60 s. Calibration solutions (0–5 μg/L Mn ions) were prepared by diluting control plant digests 100-fold to ensure matrix matching. The transport efficiency of sP-ICP-MS in enzyme-digested plant samples was established as 6.7% using monodisperse 56 nm gold nanoparticles. Additional instrument conditions and settings are provided in Table S3.

### 2.9. Statistical Analysis

The statistical analysis was conducted with SPSS 25.0, employing one-way ANOVA to assess the data. Significance levels were determined at *p* ≤ 0.05, with Duncan’s test applied for mean comparisons. Each experimental condition was tested in triplicate to ensure reliability. All experiments had six replicates except for sp-ICP-MS, which was performed in triplicate.

## 3. Results

### 3.1. Characterization of Mn-Based NMs

**Figure 1:**
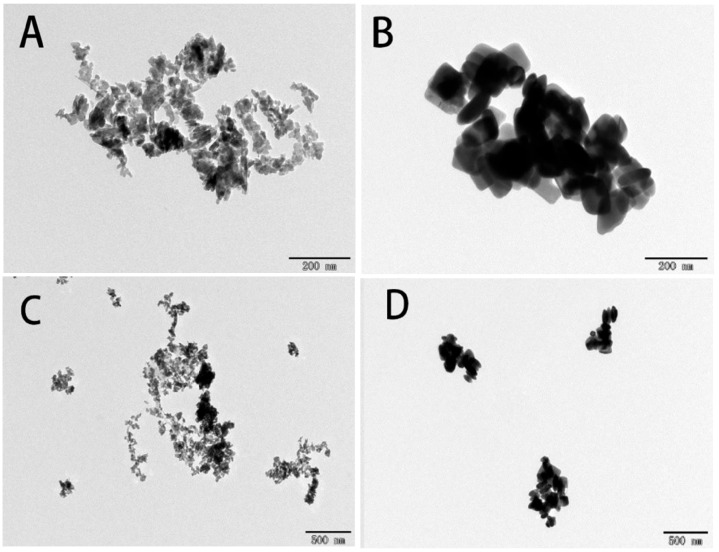
TEM images of Mn-based NMs. MnO_2_ NMs (**A**,**C**), Mn_3_O_4_ NMs (**B**,**D**).

The average sizes (*n* = 3) were 32 ± 6 nm and 77 ± 14 nm for MnO_2_ and Mn_3_O_4_, respectively (Figure 1). The hydrodynamic sizes and zeta potential in deionized water were characterized by using a Malvern Zetasizer (Nano ZS90, Malvern, UK). The hydrodynamic sizes were 387 ± 12 nm and 788 ± 7 nm, and the zeta potential for MnO_2_ and Mn_3_O_4_ were 13 ± 0.5 mv and −4.38 ± 0.1 mV, respectively.

### 3.2. Physiological Indicators of In Vivo Inoculated Rice Treated with Mn-Based NMs

**Figure 2:**
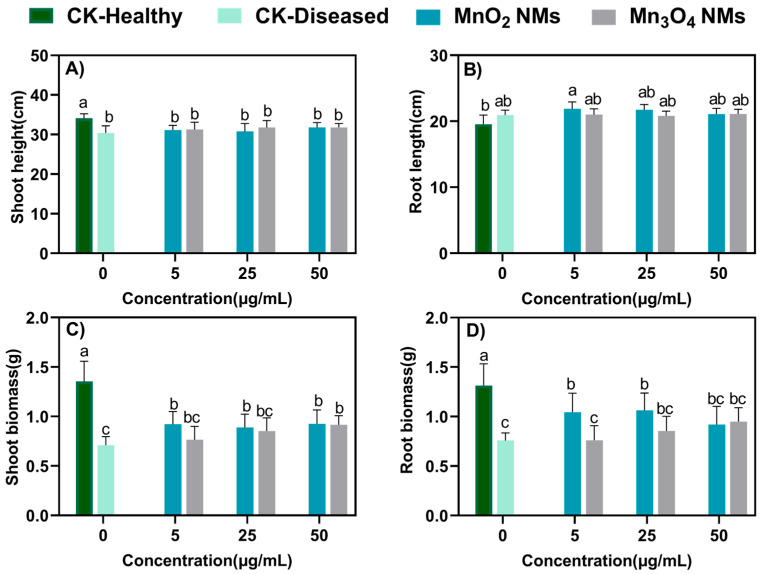
Physiological indicators of Xoo-infected rice treated with MnO_2_ NMs and Mn_3_O_4_ NMs. (**A**) rice height for all treatments, (**B**) root length; (**C**) shoot biomass; (**D**) root biomass. Columns marked with the same letter were not significantly different at *p* < 0.05 (*n* = 6).

The same range of 5–50 mg/L, which is not harmful to rice growth according to previous research [39], was chosen for the antimicrobial experiment of foliar spraying, since Xoo usually attacks rice leaves. We defined the control group grown hydroponically without bacterial inoculation as CK-Healthy, and the control group grown hydroponically under normal conditions after bacterial inoculation as CK-Diseased. The presence of Xoo caused significant damage to rice seedlings, resulting in a 10%, 48% and 44% decrease in rice height, aboveground biomass and underground biomass, respectively. In addition, aboveground biomass was significantly increased by 13%, 15% and 10% in the 5, 25 and 50 μg/mL MnO_2_ NMs treatment groups, respectively, compared to the CK-Diseased group (Figure 2). The aboveground biomass increased in a dose-dependent manner in the Mn_3_O_4_ NMs treatment group, which was significantly higher than the CK-Diseased group at 50 μg/mL. In addition, the contact angle of Mn-based NMs solution with the leaf surface was smaller than that of water with the leaf surface, indicating that the Mn-based NMs solution has better wettability and infusibility (Figure S1). Moreover, the stability of Mn_3_O_4_ NMs was higher. This suggests that the Mn-based NMs solution can adhere more effectively to the leaf surface, thereby increasing the contact area with the leaf surface, which is conducive to the distribution and absorption of nanomaterials on the leaf surface, and enhances the antibacterial effect and plant protection ability of the material. This characteristic can enhance the application potential of Mn-based NMs as nano-pesticides and improve their efficiency in plant protection.

### 3.3. Effect of Mn-Based NMs Treatment on Rice Infected by Xoo

**Figure 3:**
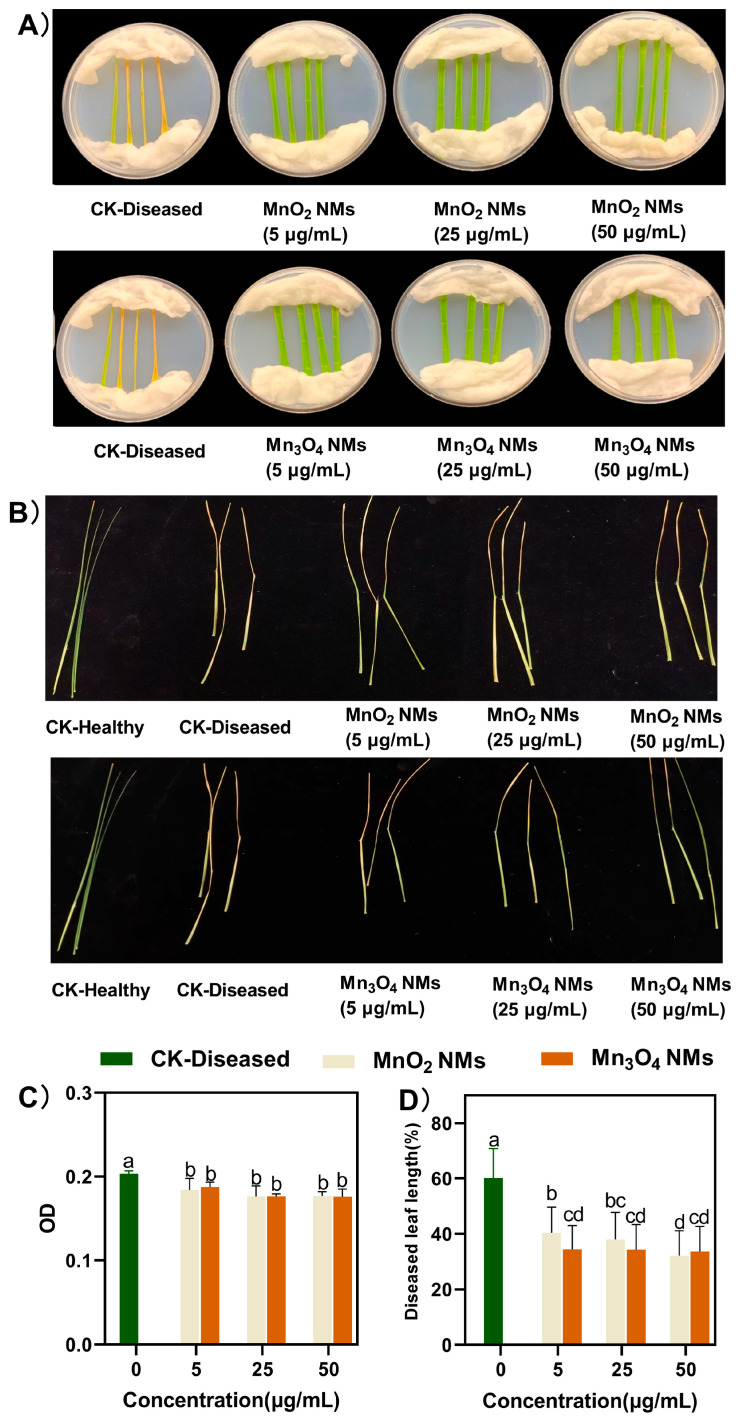
In vitro and in vivo antimicrobial experiments with MnO_2_ NMs and Mn_3_O_4_ NMs. (**A**) the rice leaf for the in vitro antimicrobial experiment, (**B**) the leaf for the in vivo antimicrobial experiment; (**C**) the OD value for the in vitro antimicrobial experiment; (**D**) the length of rice leaf susceptible to Xoo for the in vivo antimicrobial experiment. Columns marked with the same letter were not significantly different at *p* < 0.05 (*n* = 6).

Mn-based NMs have excellent antimicrobial activity at low concentrations and therefore have great potential as an antimicrobial agent to control plant diseases [40]. The in vitro antibacterial experiments showed that the leaves of rice seedlings were significantly dried, curled, and yellowed in the CK-Diseased, while the leaves of the group sprayed with Mn-based NMs grew well, with no other adverse symptoms on the overall leaves except for a small number of bacterial spots at the inoculum (Figure 3A). Ocsoy et al. [41] designed a formula to calculate the severity of the disease. The size of the infection points at the wound surface, which accounted for less than 5% of the leaf area, could be considered non-infection. Since the DLA of susceptible leaves in the isolation experiments was less than 5%, it can be concluded that spraying Mn-based NMs pairs can reduce the extent of Xoo infection in rice. The results shown that both Mn-based NMs treatments significantly reduced DLA in a dose-dependent manner compared to the CK-Diseased group. In vivo experiments, spraying MnO_2_ NMs and Mn_3_O_4_ NMs reduced the diseased leaf area to 22–28% and 25–26%.

The antibacterial activity of different concentrations of Mn-based NMs against Xoo was evaluated by measuring the OD value of the bacterial suspension. As shown in Figure 3, the OD value of the bacterial solution was 0.1 prior to incubation, and after 24 h of incubation, the concentration of the control bacterial solution was significantly higher than that of the NMs-treated solution. This indicated that different concentrations of Mn-based NMs could inhibit the growth of Xoo; the higher the concentration the better the inhibition effect. At 50 μg/mL, the OD values of MnO_2_ NMs and Mn_3_O_4_ NMs were 0.177 and 0.175, respectively (the OD value of CK was 0.23). Similarly, Gao and Keller [42] also demonstrated that high concentrations of NMs can reduce OD values. This may be because metal ions released from NMs can interfere with cellular metabolism and disrupt cell structure by binding to membranes, enzymes, nucleic acids and other cellular components. In addition, this may be because NMs inhibit biofilm activity and swimming ability, which reduce the reproduction rate of pathogenic bacteria [43]. It was shown that NMs can affect the regulatory circuits involved in pathogen motility and biofilm formation. On the one hand, they can inhibit the multiplication of pathogens and slow down their population growth, and on the other hand, they can disrupt the ability of pathogens to infect their hosts, thus they can be an alternative to traditional antimicrobial agents [44].

### 3.4. Antioxidant System of Rice Infected by Xoo

Phenylalanine amino lysin (PAL) is a key enzyme that contributes to the induction of salicylic acid (SA) synthesis, which causes systemic resistance in plants. PAL is known to be rapidly up-regulated in response to fungal infection [45]. For the above-ground part, PAL activity increased to different degrees for each treatment compared to CK-Diseased. In the treatments of 5, 25, and 50 ug/mL MnO_2_, PAL activity increased by 42%, 61%, and 41%, respectively. For plants treated with Mn_3_O_4_ NMs, PAL activity initially increased and subsequently decreased. Both Mn-based NMs maximized PAL activity at 25 ug/mL. Similarly, Ma et al. [46] found that, when tomatoes were infested with *Fusarium*, foliar application of nanoscale hydroxyapatite (nHA) at both low and high concentrations, regardless of particle size, enhanced PAL activity by approximately 30% to 80%, thereby helping to alleviate the induced stress.

The oxidative enzymes PPO and POD play crucial roles in plant defense mechanisms against biotic stresses [47]. For the aboveground parts of rice, the POD activity of all treatments was higher than that of the group CK-Diseased. The maximum enzyme activity of the MnO_2_ NMs treated group was 25 ug/mL while the maximum enzyme activity of the Mn_3_O_4_ NMs treated group was 50 ug/mL. In addition, the Mn_3_O_4_ NMs treated group significantly increased the POD activity of the roots by 144–213%. Recently, Ahmed et al. [48] sprayed bioengineered chitosan iron nanocomposites onto Xoo-infected rice, resulting in a 38.8% increase in leaf POD activity.

**Figure 4:**
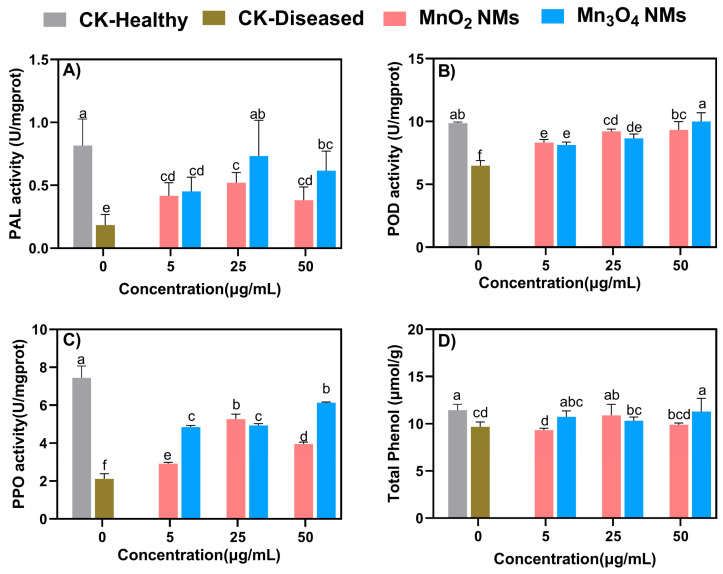
PAL activity (**A**), POD activity (**B**), PPO activity (**C**), and total phenolic content (**D**) of rice shoot for each treatment group exposed to different concentrations of MnO_2_ NMs and Mn_3_O_4_ NMs. Columns marked with the same letter were not significantly different at *p* < 0.05 (*n* = 6).

Plant phenolic compounds, as non-enzymatic antioxidants, play a significant role in secondary metabolic processes. The trend of the total phenol content was consistent with that of the PPO activity in the Mn-based NMs treatment. For the aboveground parts, the content of total phenol increased by 11% under 25 μg/mL of MnO_2_ NMs treatment. At the same time, the total phenol content increased under Mn_3_O_4_ NMs treatment. For the roots, the total phenolic content showed a decreasing trend under the treatment of MnO_2_ NMs but an increasing trend under the treatment of Mn_3_O_4_ NMs (Figure 4). Similarly, Ma et al. [46] reported that diseased plants showed a 13–60% increase in phenolic content in shoots and a 7–24% increase in roots after exposure to nHA of varying sizes.

Mn-based NMs alleviate a range of oxidative stress responses brought about by Xoo by enhancing the activity of rice antioxidant system enzymes. In addition, some NMs have certain enzyme-like activities that trap free radicals to protect plants [49]. For example, Wu et al. [50] demonstrated that CeO_2_ NPs enhance ROS scavenging and photosynthesis of *Arabidopsis thaliana* plants under excess light.

### 3.5. Accumulation of Mn-Based NMs and Ionic Mn Revealed by Sp-ICP-MS

**Figure 5:**
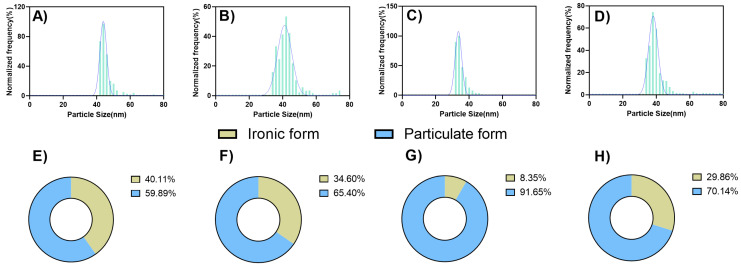
The particle size distribution of MnO_2_ NMs in (**A**) shoots, (**B**) roots; Mn_3_O_4_ NMs in (**C**) shoots, and (**D**) roots, was measured by sP-ICP-MS. The formal composition (ironic and particulate forms) of MnO_2_ NMs in (**E**) shoots, (**F**) roots; Mn_3_O_4_ NMs in (**G**) shoots, (**H**) roots. Data are means ± standard deviations of 3 replicates.

To confirm the migration and accumulation of Mn-based NMs and Mn ions in plants, sp-ICP-MS was used to identify the content of Mn-based NMs in different parts of rice. The narrower particle size distributions were obtained in the aboveground parts of rice (Figure 5A,C), with median diameters of 45 nm and 30 nm under MnO_2_ NMs and Mn_3_O_4_ NMs treatments, respectively, indicating that leaves preferentially absorbed smaller size NMs. This is consistent with the morphological composition diagram of Mn, where the smaller the absorbed NMs, the less the ionic state transition. Previous studies have also shown that NMs have unique properties compared to ions [51].

The biomass data obtained from rice indicate a more pronounced antimicrobial activity for Mn_3_O_4_ NMs relative to MnO_2_ NMs. This enhanced bioactivity likely arises from the higher stability and persistence of Mn_3_O_4_ NMs within plant tissues, predominantly maintained in nanoparticle form rather than transitioning into ionic states. In contrast, MnO_2_ NMs demonstrate a greater propensity for ionic dissolution, potentially diminishing their effectiveness in antimicrobial applications.

Particle size distributions within root tissues showed considerably larger median diameters (43 nm and 38 nm for MnO_2_ NMs and Mn_3_O_4_ NMs, respectively) compared to aboveground tissues. This finding is consistent with plant physiological limitations, as larger nanoparticles are generally restricted in uptake by plant roots.

## 4. Discussion

The findings from this study highlight the antimicrobial efficacy of Mn-based NMs (Mn_3_O_4_ and MnO_2_) against Xoo, and their potential to simultaneously promote plant growth. The observed improvement in disease resistance and biomass are consistent with previous studies that have demonstrated the efficacy of NMs as antimicrobial agents and growth promoters. For example, MoS_2_-Cu NMs have been shown to form protective layers on the leaf surfaces, increasing the capillary density and preventing Xoo infection [33]. Similarly, the ability of Mn_3_O_4_ NMs to enhance the surface stability was demonstrated in a study by Rebora et al. (2023) [52], who reported that ZnO NMs improve adhesion to the plant surface, enhancing their antimicrobial properties and reducing bacterial infections. This property is key for ensuring antimicrobial activity, making Mn_3_O_4_ a promising candidate for nanopesticide development in agricultural systems.

Furthermore, the antimicrobial mechanisms observed in this study are consistent with previous research showing that NMs can disrupt bacterial cellular functions such as membrane integrity, metabolism, and biofilm formation. For example, Ahmed et al., (2022) [48] demonstrated that chitosan–iron nanocomposites effectively inhibited Xoo by impairing bacterial motility and respiratory enzymes. Similarly, our study suggests that Mn-based NMs induce the generation of ROS within bacterial cells, leading to oxidative stress and subsequent cellular damage. Abdallah et al. (2020) [53] showed that the interaction between NMs and bacterial cells leads to ROS generation, disrupting the bacterial respiratory chain and causing cell death. The enhanced plant defense mechanisms observed in this study, particularly the increased activity of antioxidant enzymes (PAL and POD), are proved by previous research of Schiavi et al. (2023b) [54], who observed that NMs, such as CuO and Ag, could activate the plant immune system, leading to enhanced resistance against fungal infections. This further supports the idea that NMs not only serve as antimicrobial agents but also activate broader plant defense mechanisms, improving overall plant health and resilience. Additionally, the significant accumulation of phenolic compounds in the treated plants suggests that Mn-based NMs may also stimulate the secondary metabolism of plants, a phenomenon observed in several studies involving other NMs. Choudhary et al. (2019) demonstrated that NMs increased the phenolic content in plants, contributing to enhanced stress tolerance and resistance to pathogens [55]. In this study, the increase in non-enzymatic antioxidants, such as phenolic compounds, reflected the increased ability of plants to resist oxidative stress, a key mechanism for increasing resistance to biotic and abiotic stresses.

The results from sp-ICP-MS analysis provide valuable insights into the bioavailability and distribution of Mn_3_O_4_ and MnO_2_ NMs within plants, highlighting their differential mobility and transformation dynamics. Both Mn_3_O_4_ and MnO_2_ NMs were successfully taken up by plant tissues, but Mn_3_O_4_ exhibited superior stability and smaller particle size, which facilitated greater persistence and enhanced antimicrobial bioactivity within the tissues. Our previous research demonstrated that smaller and more stable Mn-based NMs could increase plant uptake and improve bioactivity [39]. Additionally, Dimkpa et al. (2013) emphasized that NMs with a high surface area and reactivity, such as Mn-based NMs, enhance interactions with both plant and pathogen cells, resulting in more effective pathogen control [56]. In contrast, the higher ionic dissolution tendency of MnO_2_ NMs may limit their sustained antimicrobial efficacy. This is further supported by our observations, in which larger particle sizes and aggregation patterns were noted in root tissues, suggesting that NMs aggregation occurs post-translocation from the leaves. As NMs are absorbed through foliar pathways and migrate downward via the phloem, they become concentrated in the roots, where localized aggregation likely occurs due to higher particle density and prolonged interactions. The observed differences in aggregation behavior between MnO_2_ and Mn_3_O_4_ NMs could significantly influence their mobility, accumulation, and overall bioactivity within the plant. This underscores the importance of evaluating the stability and transformation dynamics of NMs to optimize their application in agricultural and environmental contexts. In contrast, the higher ionic dissolution tendency of MnO_2_ NMs in solution might limit their sustained antimicrobial efficacy. Previous research proved that the dissolution of NMs could release toxic ions, potentially reducing their antimicrobial effects over time [57]. Similarly, the aggregation of larger NMs in root tissues, observed in this study, may also reduce their mobility and overall effectiveness [58].

In addition, Dinesh et al. (2012) [59] emphasized the importance of assessing the ecological risks associated with the use of NMs in agriculture, including their potential toxicity to beneficial soil microorganisms and their persistence in the environment. Several studies have reported potential phytotoxic effects of Mn-based NMs when applied at high concentrations, such as inhibited root elongation and reduced chlorophyll content in wheat and lettuce seedlings [60], and significant disruptions in soil microbial community structure and enzyme activities at elevated NMs doses [61]. These findings underscore the necessity of dose optimization and thorough toxicity assessments to ensure environmental safety when applying Mn-based NMs in agricultural systems. Such considerations are critical in understanding the broader implications of using Mn-based NMs in agricultural practices. Furthermore, while the antimicrobial efficacy of Mn_3_O_4_ NMs was clearly demonstrated in this study, the effects of these NMs on other plant diseases and pathogens warrant further exploration. Omran and Baek (2022) [62] suggested that different types of NMs may exhibit varying degrees of efficacy against different pathogens, highlighting the need to investigate whether Mn_3_O_4_ NMs can be effective against a broader range of plant diseases.

## 5. Conclusions

In this study, the inhibitory effects of MnO_2_ and Mn_3_O_4_ nanomaterials (NMs) on rice leaf blight were examined. Low concentrations of these Mn-based NMs enhanced the activity of PAL, PPO, and POD, thereby inhibiting the growth of Xoo and improving rice resistance. Notably, the Mn_3_O_4_ NMs, which were more particulate in form compared to the MnO_2_ NMs, induced higher PAL and PPO activities. As a novel nanopesticide, Mn-based NMs exhibit strong antibacterial properties, delivering significant antibacterial effects at low dosages with minimal biological damage. Despite these promising findings, there are several limitations to this study that warrant further investigation. First, the long-term environmental impact of Mn-based NMs remains unclear, particularly with regard to their potential accumulation in soil and effects on non-target organisms. Future research should include a more comprehensive toxicity evaluation to assess the potential environmental risks of Mn-based NMs, as well as their effects on soil health and biodiversity. Moreover, while the antimicrobial efficacy of Mn_3_O_4_ NMs was clearly demonstrated in this study, the effects of these NMs on other plant diseases and pathogens should be explored.

## Data Availability

The original contributions presented in this study are included in the article/Supplementary Material. Further inquiries can be directed to the corresponding author.

## Supplementary Materials

The following supporting information can be downloaded at: https://www.mdpi.com/article/10.3390/plants14101540/s1, Figure S1: Contact angle pictures of rice leaves exposed to 0, 5, 25, 50 μg/L of MnO_2_ NMs (A–D), 5, 25, 50 μg/L Mn_3_O_4_ NMs (E–G); and contact angle numbers (H); Figure S2: The concentrations of MnO_2_ NMs (50 mg/kg) in rice (A) shoots and (B) roots; and Mn_3_O_4_ NMs in rice (C) shoots and (D) roots were determined by single particle inductively coupled plasma mass spectrometry (sP-ICP-MS) Table S1: Zeta potential and hydrodynamic diameter properties of Mn-based NMs. Data presented are mean ± STDEV (n = 3); Table S2: Formulation of 1 L NA liquid medium; Table S3: The composition of Kimura nutrient solution; Table S4: Single Particle ICP-MS Instrumental Parameters.
