# Supplementary material for: Manganese Nanomaterials: A Green Solution to Suppress Xanthomonas oryzae in Rice
Source: Plants (Basel). 2025 May 20;14(10):1540. doi: 10.3390/plants14101540 (PMC12114837; doi:10.3390/plants14101540)
Supplement: Supplementary file 1 [file plants-14-01540-s001.zip › plants-3564029-supplementary.pdf]

Supporting Information

Manganese based nanomaterials suppress rice bacterial blight pathogen *Xanthomonas oryzae*

Yaqi Jiang, Yi Sun, Pingfan Zhou, Meng Tian, Yukui Rui

Supplementary information:

Number of pages: 7

Number of Supplementary Figures: 2

Number of Supplementary Tables: 4

Figure:

Figure S1 Contact angle pictures of rice leaves exposed to 0,5,25,50 μg/L of MnO_2 NMs (A)(B)(C) and (D), 5,25,50 μg/L Mn_3O_4 NMs (E)(F) and (G); and (H) contact angle numbers

Figure S2 The concentrations of MnO_2 NMs (50 mg/kg) in rice A) shoots and B) roots; and Mn_3O_4 NMs in rice C) shoots and D) roots were determined by single particle inductively coupled plasma mass spectrometry (sP-ICP-MS)

Table:

Table S1 Zeta potential and hydrodynamic diameter properties of Mn-based NMs.

Table S2 Formulation of 1 L NA liquid medium

Table S3 The composition of Kimura nutrient solution

Table S4 Single Particle ICP-MS Instrumental Parameters

Section 1. Methods

Text S1. Determination of contact angle

Contact angle observation to assess the efficiency of the use of Mn-based NMs in relation to particle size, the contact angle of two types of Mn-based NMs in rice leaves was measured using an optical tensiometer (Biolin Scientific) (Huang et al., 2017). Each Mn-based NMs was measured three times.

Section 2. Results

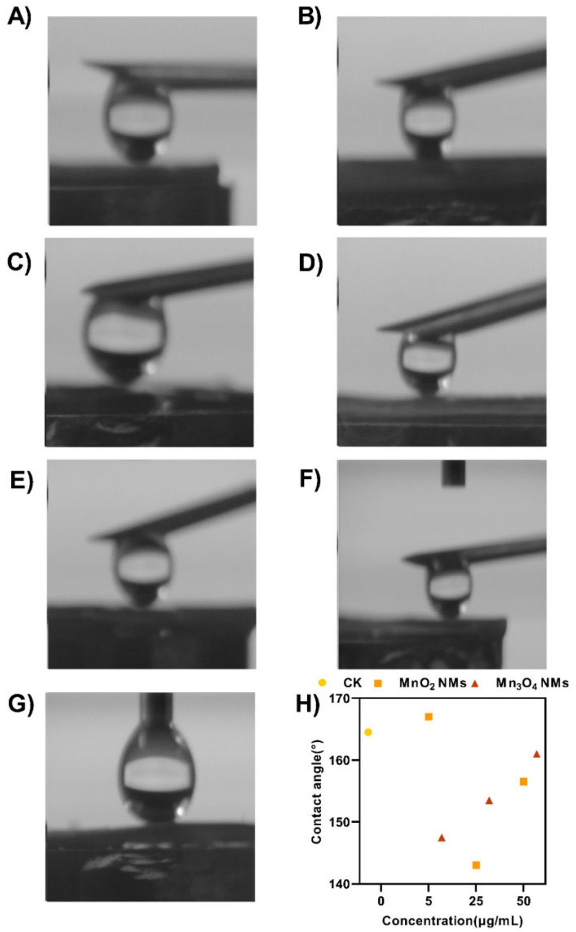

Figure S1 Contact angle pictures of rice leaves exposed to 0,5,25,50 μg/L of MnO_2 NMs (A)(B)(C) and (D), 5,25,50 μg/L Mn_3O_4 NMs (E)(F) and (G); and contact angle numbers (H).

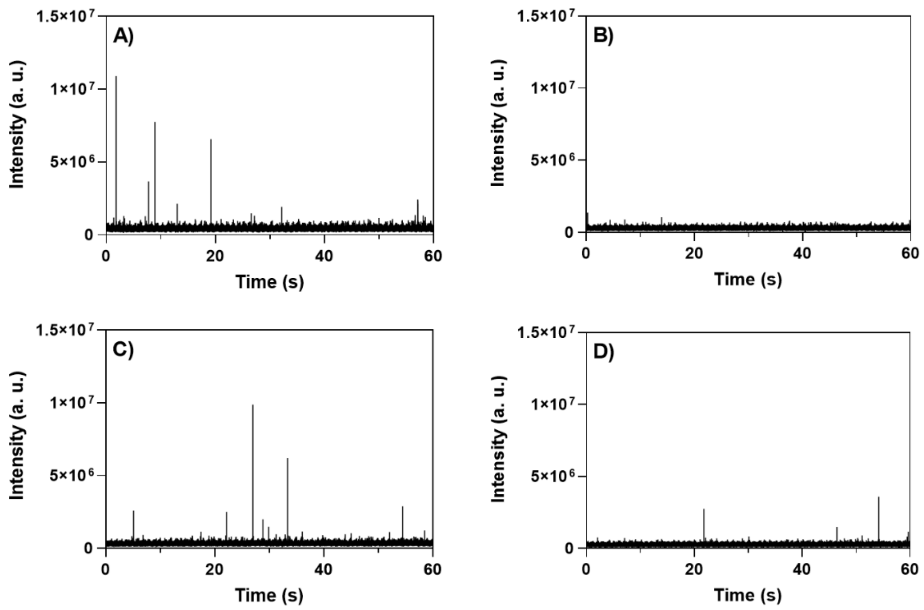

Figure S2 The concentrations of MnO_2 NMs (50 mg/kg) in rice A) shoots and B) roots; and Mn_3O_4 NMs in rice C) shoots and D) roots were determined by single particle inductively coupled plasma mass spectrometry (sP-ICP-MS)

Table S1. Zeta potential and hydrodynamic diameter properties of Mn-based NMs. Data presented are mean ± STDEV (n =3).

	MnO_2 NMs	Mn_3O_4 NMs
Size(nm)	32±6 nm	77±14 nm
Hydrodynamic diameter(nm)	387±12	788±7
zeta(mV)	13±0.5	-4.38±0.1

Table S2 Formulation of 1 L NA liquid medium

**Complement**	**Content(g)**
Beef extract	3.0
Peptone	5.0
Yeast powder	1.0
Cane sugar	10.0

Table S3 The composition of Kimura nutrient solution

Chemicals	Concentrations
$Ca(NO_3)_2$	0.37 mM
KNO_3	0.18 mM
$(NH_4)_2SO_4$	0.37 mM
Fe(II)-EDTA	0.05 mM
$CuSO_4$	0.001 mM
NaCl	0.1 mM
H_3BO_4	0.01 mM
$MgSO_4$	0.55 mM
KH_2PO_4	0.18 mM
K_2SO_4	0.09 mM
$ZnSO_4$	0.001 mM
$MnSO_4$	0.005 mM
Na_2MoO_4	0.0005 mM
$CoSO_4$	0.0002 mM

Table S4 Single Particle ICP-MS Instrumental Parameters

Parameters	Value
Sample Uptake Rate	0.307 mL/min
Nebulizer gas flow	1.05 L/min
Nebulizer	Meinhard Glass Concentric
Spray Chamber	Meinhard Glass Cyclonic
RF Power	1550 W
Analyte	^{59}Mn
Analysis time	60 s
Dwell time	100 µs
Analyte monitored	^{197}Au
Transport efficiency	6.3%

Table S4 Abbreviation

Abbreviation	Full name
MnO_2	Manganese oxide
NMs	Nanomaterials
Mn_3O_4	Manganese tetroxide
Xoo	*Xanthomonas oryzae*
PAL	Phenylalanine ammonia lyase
BLB	Bacterial leaf blight
POD	Peroxidase
SOD	Superoxide dismutase
CAT	Catalase
MDA	Malondialdehyde
NA	Nutrient agar
DLA	Disease leaf area
PPO	Polyphenol oxidase
O_2	Molecular oxygen
K	Potassium
P	Phosphorus
Ca	Calcium
Mg	Magnesium
Fe	Iron
Cu	Copper
SA	Salicylic acid
